# Neurological potency of native plants from sub-Himalayan West Bengal through reverse pharmacology

**DOI:** 10.6026/97320630019995

**Published:** 2023-10-31

**Authors:** Lepcha Tshering Dhendup, Datta Sutapa, Joongku Lee, Ajmal Ali Mohammad, Arnab Sen

**Affiliations:** 1 Molecular Genetics Laboratory, Department of Botany, University of North Bengal, Raja Rammohanpur, Siliguri-734013, India; 2 Bioinformatics Facility Centre, University of North Bengal, Raja Rammohanpur, Siliguri-734013, India; 3 Biswa Bangla Genome Centre, University of North Bengal, Raja Rammohanpur, Siliguri-734013, India; 4 Department of Environmental and Forest Resources, Chungnam National University, Daehak-ro, Yuseong-gu, Daejeon, Republic of Korea; 5 Department of Botany and Microbiology, College of Sciences,King Saud University, Riyadh 11451, Saudi Arabia

**Keywords:** Neurological potency, native plants, sub-Himalayan West Bengal, reverse pharmacology

## Abstract

Neurodegenerative diseases, such as Alzheimer's disease (AD), Parkinson's disease (PD), and epilepsy, pose a growing global health challenge due to an aging population. These conditions share common processes, including protein accumulation, oxidative stress, and neuro-inflammation, making their treatment complex and costly. Network pharmacology, an innovative approach integrating systems biology and computational biology, offers insights into multi-target formulations and the repurposing of existing medications for neurodegenerative diseases. We shortlisted 730 bioactive compounds from 25 traditional Himalayan plants, assessed their drug-like properties using ADME criteria, and predicted their potential target proteins through reverse docking and pharmacophore mapping. Our study identified 287 compounds with high gastrointestinal absorption and good blood-brain barrier permeability. These compounds were subjected to target prediction, yielding a list of 171 potential target proteins. Functional annotation and pathway enrichment analysis highlighted their involvement in steroid hormone-related pathways, MAPK signaling, FOXO signaling, TNF signaling, VEGF signaling, and neurotrophin signaling. Importantly, one plant, *Valeriana jatamansi*, exhibited an association with beta-amyloid binding activity, a potential therapeutic approach for AD. From our study we could understand how these plants modulate our body to manage these diseases. However, further in vitro and in vivo validation is needed before commercial and public use of this data.

## Background:

Neurodegenerative diseases are the progressive deteriorating conditions characterized by the death of nerve cells and supreme loss of neurons. Neurodegenerative diseases are age-related conditions that have an effect on the brain as well as the nerves found throughout the human body and the spinal cord. These age-dependent disorders have become progressively rife, partly as a result of the senior population has risen in recent years. Neurodegenerative diseases mainly include Alzheimer's disease (AD), Parkinson's disease (PD), Huntington's disease (HD), Amyotrophic lateral sclerosis (ALS), front temporal dementia and the spinocerebellar ataxias [1]. Neurodegenerative diseases are generally outlined by specific macromolecule accumulations and anatomic vulnerability. Neurodegenerative diseases share several basic processes related to progressive neuronal dysfunction and death, such as proteotoxic stress and its attendant abnormalities in ubiquitin proteasomal and autophagosomal/lysosomal systems, oxidative stress, programmed cell death, and neuro-inflammation [2]. In addition to aging, susceptibility to genetic and environmental factors increases the risk of being affected by these diseases [1].

Among all, presence of AD is considerably high and is increasing per annum. The global burden of AD is anticipated to accelerate from 26.6 million cases in 2006 to 106.8 million by 2050 [3]. AD is characterized by language impairment, progressive memory loss, odd behaviour, cognitive decline, personality and mood changes, as well as disability to perform daily activities, eventually turning fatal. Neuropathologically, AD is characterized by brain region-specific deposition of β-amyloid protein (Aβ) which creates senile plaques, hyper-phosphorylation of the cytoskeletal protein Tau that forms lesions called neurofibrillary tangles and neuropil threads, glial activation which is associated with inflammatory responses, and both synaptic and neuronal loss [4]. Also, PD is a brain disorder that leads to shaking, stiffness, and difficulty with walking, balance, and coordination. It is also progressive in nature [1]. Another common disorder, epilepsy, is a central nervous system (neurological) disorder in which brain activity becomes abnormal, causing seizures or periods of unusual behaviour, sensations and sometimes loss of awareness [5]. It is an uncommon comorbidity of PD and has been considered not directly associated with PD. However, people with epilepsy develop Alzheimer's disease at a rate 6 times higher than the non-epileptic population and people with epilepsy are at a substantially increased risk of developing dementia [5].

Unfortunately, the complex etiology and pathogenesis of neurodegenerative diseases limit the efficacy of drugs for their treatment [1], and high expenditures results in deprivation of proper treatment in most cases. In this context, initiatives such as network pharmacology may provide new directions for curing these neural diseases. It is a pioneering method that includes systems biology and computational biology, which utilises multi-component and multi-target formulations. This aids in the understanding of host and drug interactions and helps in the repurposing of existing medicines [6]. It also provides a better platform for understanding the mechanism of action of the bioactive compounds identified from traditionally used plants.

Here, we studied twenty-five Himalayan plants mainly from Darjeeling and some Sikkim region which are traditionally used treating neural diseases like epilepsy, headache, and other CNS related diseases. Traditionally many plants are used in different regions by local healers to treat many of these neurological disorders. The sub-Himalayan region of Eastern-India is also rich in such traditional knowledge. Darjeeling, the northern most part of the state West Bengal, India, is situated in the foothills of the Himalayas. It is rich in culture and is inhabited by different tribes. These tribes have treated diseases like epilepsy, headache and other neurological disorders using plants such as *Cannabis sativa*, *Cyperus rotundus*, *Ravoulfia serpentina*, and many others [7,8,9, 10,11]. Plants generally help in disease management through regulation of numerous proteins in human body. Rather than interacting with a single specific protein, the multiple phytochemicals present in plants may modulate multiple protein targets and pathways of our body. Such management of diseases by these plants may be understood with the aid of network pharmacology [6]. Therefore, it is of interest to explore the efficacy of ethno-medicinally used plants from Darjeeling Hills against neurological disorders through network pharmacology that will aid in understanding the mechanism of action of these plants. This may also shed light on lesser known drug candidates and new therapeutic pathways in neurological disease treatment.

## Methodologies:

## Selection of Plants and their chemical constituents:

An extensive study was done to understand the traditionally used plants of Darjeeling Hills and Sikkim which are used for treating neural diseases like epilepsy, headache and other CNS related diseases. Along with literature survey, we also talked with some of the locals of the region and validated the documented knowledge. A total of 25 plants from Himalayan region which are traditionally used in treating neural disease were selected using literature study. The bioactive compounds of the selected plants were obtained from Dr. Duke's Phytochemical and Ethnobotanical database [12]. Dr. Duke provides in-depth information on plants, their chemical constituents, bioactivities and ethnobotany.CID number (Compound identification number) and canonical smiles were obtained from the database PubChem (https://pubchem.ncbi.nlm.nih.gov/) for further analysis.

## Drug-ability analysis of the phytocompounds:

The drug likeliness of the phytocompounds is determined on the basis of their ADME property. ADME (absorption, distribution, metabolism, and excretion) property evaluation helps to get an idea regarding how a chemical is processed by a living organism [13]. Thus, with the help of Swiss-ADME [13], it was assessed whether the compounds abide by rules such as "Lipinski's rule of five", "Veber's rule", etc. Also, the presence of any PAINS (pan assay interference compounds) was determined. The SMILES formula (a computer friendly representation of chemical structures) of each compound was submitted to the server for this analysis. The treatment of CNS diseases is particularly difficult since the BBB restricts the access of many compounds to their CNS targets [14]. Thus, for further analysis, only the compounds showing both Blood-Brain Barrier (BBB) permeability and Gastrointestinal (GI) absorption were taken into consideration.

## Target prediction of the selected compounds and network construction:

The phytocompounds interact with numerous proteins of our body and modulate them. PharmMapper predicts potential target candidates for the given small molecules on the basis of reverse docking and pharmacophore mapping approach [15]. The selected chemical compounds were submitted in SDF (Structure Data Files) format and we opted for "Human Protein Targets Only". The target proteins were then sorted out on the basis of normalized fit score (≥ 0.9) and z-score (≥ 1). Large positive z-score indicates high significance of the target to a query compound. Only the plants with target proteins falling under these criteria were taken into consideration. This data was used to create a phytocompound-target network with the help of Cytoscape v3.8.2 [16].

## Functional annotation and KEGG pathway enrichment analysis:

To understand the role of a set of genes in metabolic pathways, gene ontology analysis is important. The target-gene list of each plant was submitted in DAVID Bioinformatics Resources v6.8 (https://david.ncifcrf.gov/tools.jsp) for functional annotation and KEGG pathway enrichment analysis. It gives an insight in role of these proteins in different metabolic pathways. Gene ontology (GO) enrichment analysis comprised Biological Process (BP), Cell Component (CC), and Molecular Function (MF). The results with significant p-Value (p-value ≤ 0.05) were taken into consideration.

## Results and Discussion:

A total of 25 plants from Himalayan region which are traditionally used in treating neural disease were selected using literature study. The plant list is provided in the supplementary file.

## Phytochemical constituent and their drug likeliness:

**Figure 1:**
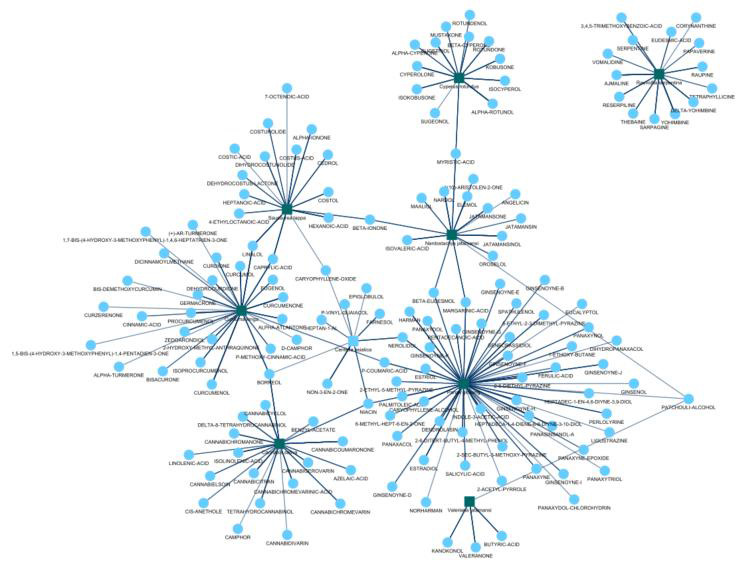
Plant-phytocompound interaction network. The green squares denote the plants and the blue circles are the associated phyto-compounds.

Total 1362 phytochemical compounds were obtained from the studied plants, out of which 730 compounds having canonical smiles and CID number. The ADME properties of the acquired compound were analyzed, which shows 287 compounds with High Gastrointestinal Absorption (GI) and good Blood Brain Barrier (BBB), 34 compounds with low Gastrointestinal (GI) absorption but good Blood Brain Barrier (BBB) permeability, 139 Compounds with high Gastrointestinal absorption (GI) but no Blood Brain Barrier (BBB) permeability, and 273 compounds with low Gastrointestinal absorption (GI) and no Blood Brain Barrier permeability (BBB). The compounds having both high GI and good BBB permeability were considered and further analysis was carried out. A plant phytocompound network of the compounds with best results is shown in Figure 1.

## Target prediction, Functional annotation, and pathway enrichment analysis:

**Figure 2:**
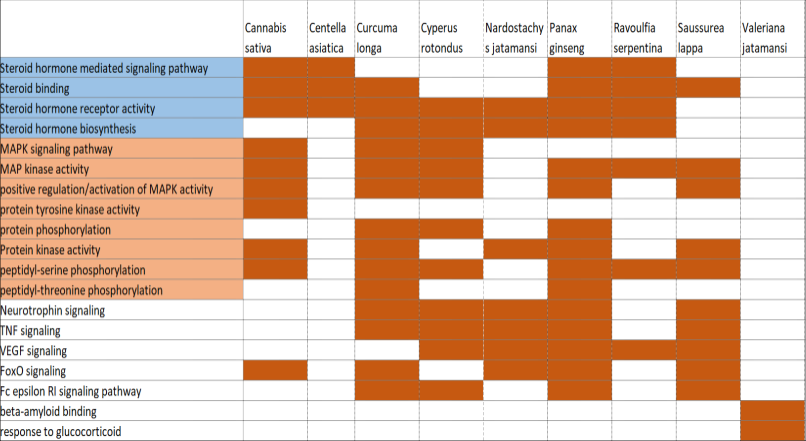
Pathways that may be modulated by selected plants in managing neural diseases. Here, a comparison between the modulatory activities of the different selected plants is shown through yellow highlights.

Target prediction of the phytocompounds of each plant gave a list of total 171 proteins. Analysis of the phytocompound-target network revealed that the plant *Panax ginseng* has the highest number of targets (51), followed by *Curcuma longa* and *Cannabis sativa*. The compound Yohimbine is the best compound as it has 15 targets. Functional annotation and pathway enrichment analysis of the targets show association of the plants with various pathways that are related to nervous system pathways in *Homo sapiens*(Figure 2).

## Modulation of steroid hormone related pathways:

The selected traditionally used plants for neural disorders (except *V. jatamansi*) were found to target several genes related to steroid hormone metabolic pathways. They show ability to modulate genes related to "steroid hormone mediated signalling pathway", "steroid hormone receptor activity", "Steroid hormone biosynthesis" and "steroid binding" activity. The plants *Centella asiatica* and *Ravoulfia serpentine* mainly act through these pathways only. *Cannabis sativa* and *Panax ginseng* too modulate a number of these pathways.

It is reported that some sex steroids, such as progesterone, act via sex steroid receptors and other more recently discovered pathways in regulation of neuronal survival and neuronal differentiation in the brain, like activity exerted by neurotrophins and classical growth factors [17]. With growing age, the levels of sex hormones decrease and this may be associated with the progression of neurodegenerative disorders, as well as increased depressive symptoms [18]. Estradiol and progesterone are also neuroprotective in experimental models of Parkinson's disease and forebrain ischaemia. Thus, the modulation of these pathways is one of the mechanisms of action employed by these plants in neural disease management.

## Control of cell signalling pathways related to neurological disorders:

Functional annotation and pathway enrichment analysis show association of the plants with MAPK signalling pathway, MAP kinase activity, activation of MAPK activity, protein tyrosine kinase activity, etc. that play role in cell proliferation. The plants *Cannabis sativa*, *Curcuma longa*, *Cyperus rotundus* and *Saussurea lappa* were mainly found to be associated with these pathways. Studies have shown that deviation from the strict control of MAPK signalling pathways have role in development of Alzheimer's disease (AD), Parkinson's disease (PD), amyotrophic lateral sclerosis (ALS) and various types of cancers [19].

Apart from MAPK signalling and kinase activity, the plants target various pathways like FoxO signaling pathway, VEGF signaling, Neurotrophin signalling pathway, etc. Foxo (Forkhead box) proteins help in regulation of growth factor and stress regulating factor. FOXO3 has been found to cause axonal degeneration upon withdrawal of neurotrophic factors and can influence both CNS and PNS [20]. Abnormal regulation of this pathway, leads to the pathogenesis of age-related diseases affecting bone, muscle, and the central nervous system [1].

The plants also modulated TNF (Tumour necrosis factor) signalling pathway and VEGF signalling pathway. Both pathways are associated with neuroprotection in the brain. Another modulated significant pathway is Neurotrophin signalling which pathway plays an important role for neural development and additional higher-order activities such as learning and memory . Thus, by regulating this pathway, development and enhancement of neural cells can be modulated, which may be helpful in preventing various neural diseases.

Modulation of all these targets and pathways may be the mode of action for these plants that have been used to treat neurological diseases from time immemorial. These may also help in finding a new approach in treating such diseases. However, in our studies it was seen that the plant *V. jatamansi* targets a different set of genes for treatment of neurological disorders, then the rest of the selected plants. Its phytocompounds interact with target proteins that have "beta-amyloid binding activity". It is known that beta-amyloid protein accumulation causes AD [1]. Thus, the association of the plant with it may be considered as a therapeutic approach. Further evaluation of the plant may shed light on a novel therapeutic approach.

## Conclusion:

In conclusion, while the human race has long been regarded as unique and superior among living organisms due to its intelligence and cognitive capabilities, the contemporary lifestyle and increasing levels of stress pose a significant threat to this exclusivity. Today's lifestyle significantly elevates the risk of various cerebrovascular diseases, potentially contributing to degenerative forms of cognitive impairment, as indicated by researchers. Neurological disorders, as defined by the World Health Organization (WHO), encompass conditions affecting both the brain and the nervous system, which extends throughout the human body. Our study sheds light on the underlying mechanisms of traditionally employed plants in addressing these issues. Notably, steroid hormones play a direct role in the aging process and the development of neural diseases, suggesting that the modulation of these pathways may be central to the action of these plants. Our findings suggest that the modulation of steroid hormone pathways and pathways related to cell viability may hold promise in managing cognitive diseases associated with neural cell damage. However, it is imperative to note that further validation through *in vivo* and *in vitro* studies is essential to gain a deeper understanding of these results and their potential implications for the management of neurological disorders.

## Author Contribution:

AS conceived the idea. TDL and SD collected data and executed the analysis. JL, MAA, SD & AS helped in data analysis using Bioinformatics tools. All the authors have contributed in writing the manuscript and approved it.

## References

[1] Slanzi Anna (2020). Frontiers in cell and developmental biology..

[2] Dugger BN, Dickson DW (2017). Cold Spring Harbor perspectives in biology.

[3] Thakur AK (2018). J anal pharm Res.

[4] DeTure MA, Dickson DW (2019). Mol Neurodegener..

[5] Zhang D (2022). Front Neurol..

[6] https://www.ncbi.nlm.nih.gov/pmc/articles/PMC7148629/.

[7] https://dspace.cus.ac.in/jspui/bitstream/1/3734/1/Some%20less%20known%20ethnomedicinal%20plants.pdf.

[8] Rai U (2008). Pleione..

[9] Chettri U, Kumari S (2021). Journal of Medicinal Plants.

[10] Rai U, Rai B (2020). Pleione.

[11] Rai KS, Bhujel RB (2012). Himaliyan Research Journal.

[12] Duke James A. (1992). Database of phytochemical constituents of GRAS herbs and other economic plants..

[13] Daina A (2017). Sci Rep..

[14] Cascione M (2020). Front Bioeng Biotechnol..

[15] Liu X (2010). Nucleic Acids Res..

[16] Shannon P (2003). Genome Res..

[17] Azcoitia I (2003). Aging Cell..

[18] DonCarlos LL (2009). Psychoneuroendocrinology..

[19] Kim EK, Choi EJ (2010). Biochim Biophys Acta..

[20] Mitre M (2017). Clin Sci (Lond)..

